# Gender-affirming care is preventative care

**DOI:** 10.1016/j.lana.2023.100544

**Published:** 2023-06-24

**Authors:** Arjee Javellana Restar

**Affiliations:** a Department of Epidemiology, University of Washington School of Public Health, Seattle, WA, USA; b Department of Social and Behavioral Sciences, Yale University School of Public Health, New Haven, CT, USA

Mental health is a major public health crisis and has become a top priority in the United States, as anxiety and depression symptoms remain elevated compared to pre-coronavirus (COVID) pandemic rates both in the general population,^1^ and in communities of transgender and nonbinary (trans) people.^2^ Addressing mental health problems among trans people necessitates explicit programmatic and investment goals that allow the equitable provision of not just treatment, but instead, an array of both preventative and treatment tools, including the integration of gender-affirming care (GAC) services consisting of high-quality medical, surgical, and mental health services that affirm and align gender goals—and are tailored to meet the needs of trans people.

For the growing 1.6 million U.S. trans people, mental health problems are particularly pressing given the disproportionally high levels we experience. My reflexive observation as a social epidemiologist and an Asian American/Filipina trans woman who experienced fragmented mental healthcare systems, and have seen fellow marginalized trans individuals also navigate similarly, led me to embrace envisioning solutions and possibilities for current and future generations of our communities, particularly when faced with existing epidemiological data on mental health. Specifically, prevalence estimates of lifetime suicide attempts, severe psychological distress, and clinical depression are as high as 40%, 39%, and 52%—rates that are estimated to be nine, eight, and six times more than the general U.S. population at 4^.^6%, 5%, and 8^.^4%, respectively.^3^ These rates are even more pronounced, particularly with younger, Black, Indigenous, and Asian American and Pacific Islander trans communities who substantially face fragmented GAC^3^—making mental health a significant health priority.

GAC is an integral determinant of trans people’s well-being.^4^ While not all trans people seek GAC, most do. For trans youth, GAC is an unimpulsive and well-informed decision between themselves, their parents, and providers—one that prioritizes parental consent and youth’s assent to fully understand the scope of the treatment, including its timeline, risks and benefits, what is currently known and not known about the impact of treatment on other bodily/physiological functioning, as well as how such treatment may or may not fit their health needs and gender goals later in life.^5^ Other forms of GAC services ranges from affirmative counselling to hormones for youths, while surgeries is limited to adults.^5^

GAC is linked to improved quality of life and mental health among trans people.^4^^,^^6^^,^^7^ Notably, in a large match control study, use of hormones was associated with less depression, and trans people not on hormones had 4-fold increased risk of depressive disorder.^8^ Results from a prospective cohort study of U.S. trans youths showed increases in positive psychological outcomes, including positive affect and life satisfaction, and decreases in depression and anxiety symptoms after receiving 2 years of hormones—addressing the lack of longitudinal data in this area.^9^ Notably, this study also reported a total of 3.5% suicidal ideation^9^—a comparable rate to the U.S. general population rate of 4.6%.^3^ To date, no studies have reported findings that suggest GAC increases negative mental health outcomes.

Where treatment for mental health is available, prevention must also be key. Currently, all GAC services are only prescribed to trans people being treated for gender dysphoria. Given that not all trans people have gender dysphoria, and that being trans is not dependent on this diagnosis, this medical dilemma between its benefits and the prerequisite diagnosis gives rise to the following questions: How do we care for trans people’s mental health who are needing GAC but do not meet criteria for gender dysphoria? Do we wait until trans people receive a clinically significant level of distress (i.e., worsened mental health outcome) that meets clinical diagnosis before providing options for GAC? How do we provide continued GAC for trans populations who may no longer have gender dysphoria, especially aging trans populations whose gender-affirmation needs may have changed or require further maintenance or want to disengage, and whose mental health are more likely to be vulnerable over time? Moreover, how do we develop a health system GAC can be accessible without pathologizing trans people with a gender dysphoria diagnosis?

Mental health prevention can be conceptualised with three different levels of approach: primary (i.e., addressing risk and increasing protective factors), secondary (i.e., early detection), and tertiary (i.e., care management)—all of which are directions that GAC has yet to be mapped out. To start, first, in line with all relevant major medical organizations, including the American Medical Association, we must learn the published evidence that points to GAC as an integral protective factor for trans people’s mental health,^4^^,^^6^^,^^9^^,^^10^ and detail responses to address socio-ecological risk factors and root causes for negative mental health, including transphobic policies. Second, we must develop better gender-specific screening tools and protocols for detecting depression, suicidality, and anxiety, among others, that capture gender-based stressors and violence risks unique to trans populations. Third, maintaining healthier mental outcomes throughout trans people’s life course will require sustainable solutions. Self-administration of GAC, for example, is one approach to expanding access that has been successfully deployed in “real-world” settings in countries like Thailand—a country that has historically made quality and affordable GAC services available to trans people throughout the world, without hinging on clinical diagnosis requirements.^11^ Similar adaptations in the context of the U.S., like making hormones available behind the counter, providing self-administration training to trans patients, and allowing pharmacists to be able to provide counselling and administer injectable hormones, could be empowering tools when delivered within safe, supportive health systems.

Over the years, the benefits of GAC has become apparent, yet beneficiaries remain exclusive, leaving many trans people to wait until eligible for treatment at the cost of worsened outcomes. While there are established treatments available outside of GAC, preventing negative mental health outcomes before they occur requires widening the tools of prevention, and calling in medical, insurer, and policy communities to value GAC in improving mental health for trans people. To address the high prevalence of mental health problems, GAC must be synergized as part of combined preventative mental health care options and strategies.

## Contributors

Dr. Restar conceptualized, wrote, edited, and reviewed this manuscript.

## Declaration of interests

The author declares no competing interest.
